# Ambient air and noise pollution effect on cardiovascular health risk and lifestyle intervention to attenuate it: study protocol for a randomized clinical trial

**DOI:** 10.3389/fpubh.2026.1747963

**Published:** 2026-01-26

**Authors:** Regina Grazuleviciene, Sandra Andrusaityte, Audrius Dedele, Ausra Kapustinskaite

**Affiliations:** 1 Department of Environmental Sciences, Vytautas Magnus University, Kaunas, Lithuania; 2 Department of Family Medicine, Lithuanian University of Health Sciences, Kaunas, Lithuania

**Keywords:** cardiovascular risk, Mediterranean diet, metabolic syndrome, noise pollution, physical activity, randomized clinical trial, ultrafine particles

## Abstract

**Background:**

A few recent studies have associated ultrafine particulate matter (UFP) with metabolic disorders, contributing to cardiovascular disease, however, evidence is inconsistent. This study aims to investigate the causal relationship between long-term ultrafine particles (UFP) and noise exposures on cardiovascular disease and whether short-term healthy lifestyle interventions can reduce the risks of metabolic disorders.

**Methods:**

The research starts from an observational cross-sectional study which involves 1,000 randomly selected 45–64-year-old Kaunas city (Lithuania) men and women. Then a three-arm randomized healthy lifestyle trial of 180 participants is conducted to study the effects of short-term lifestyle interventions, such as promoting physical activity in green spaces and Mediterranean diet. The pollution exposure patterns and the resulting health impacts are estimated on the spatial distribution and participants home addresses. The randomized trial health endpoints will be physicians assessed before intervention on Day 1 and on Day 8 (after intervention) using 7-days monitoring sensors data, clinical survey data, clinical biomarkers of metabolic disorders, and novel cardiometabolic biomarkers. Mathematical models and advanced analytical chemistry techniques will be used to estimate the associations and the effects of the interventions.

**Discussion:**

This clinical trial with an interdisciplinary approach can provide new insights by evaluating the combined impact of UFP and noise on the indicators of metabolic syndrome and the effects of short-term lifestyle interventions, such as promoting physical activity in green spaces and healthy diets. It can strengthen the evidence base for pollution-disease associations and provide practical recommendations for reducing the burden of metabolic disorders and cardiovascular diseases.

**Clinical trial registration:**

https://www.clinicaltrials.gov/study/NCT07111208, NCT07111208.

## 1 Introduction

At present, traffic-related environmental exposure accounts for up to two-thirds of all chronic non-communicable diseases and cardiometabolic-associated health outcomes such as metabolic syndrome, obesity and diabetes (1). Moreover, environmental-related risk factors, among them air pollution, noise, and personal lifestyle habits and socioeconomic factors contribute to the increase of the global burden of cardiovascular disease (CVD), metabolic syndrome, arterial hypertension, and diabetes mellitus (2, 3). Exposure to both particulate matter (PM) and noise pollution increases the risk of developing CVD and metabolic syndrome, and their combined effect might be greater than the effect of either alone (4, 5). Current evidence suggests that the development of cardiometabolic disorders can occur through PM exposure induced chronic systemic inflammation, increased oxidative stress which have an impact the components of metabolic syndrome, such as hypertension, central obesity, and hyperglycemia (6). In Europe, approximately 500,000 premature deaths occur annually due to chronic conditions caused by air pollution. A Copenhagen study presented findings showing that even short-term exposure to UFP can trigger respiratory and cardiovascular diseases morbidity and mortality (7). However, PM and noise thresholds are not currently incorporated into clinical guidelines, though the need for UFP regulation is important to help prioritize emission control measures.

When comparing the health effects of ultrafine particle (UFP) (PM < 0.1 μm diameter) and coarse PM, the damage caused by UFP is greater due to its ability to directly penetrate lung tissue, enter the bloodstream and to the brain causing cerebral and autonomic system dysfunction (8). This leads to systemic inflammation, endothelial dysfunction, coagulation abnormalities, and oxidative stress—processes that contribute to the development of metabolic disorders and CVD (1, 9). Inhaled UFP may cause the short- and long-term disorders of some blood biomarkers and lead to the progression of cardiovascular dysfunctions in vulnerable subjects living in air-polluted areas (10). At this time, metabolic disorders biomarkers vary from traditional clinical measures to novel metabolic and inflammatory indicators that might sign early risk of cardiometabolic disorders. However, the combined impact of UFP and noise exposure levels in the pathogenesis of metabolic disorders promoting the development of CVD and metabolic syndrome remains unclear. This research strengthening the evidence base for pollution-disease associations could provide policymakers with reasonable evidence to develop and implement effective public health policies and interventions, leading to a reduction in the CVD and CMD caused by pollution.

Epidemiological studies suggest that the global prevalence of metabolic syndrome is between 12.5 and 31.4%, depending on the diagnosis criteria used (11). In Lithuania, cardiovascular diseases affect more than 40% of the population aged 45–64 and have a significant impact on public health and the healthcare system (12). Findings suggest that long-term environmental exposures, psychosocial stressors (13, 14) increase in high-calorie food intake, and low physical activity have contributed to the Global rising incidence of CVD and metabolic syndrome (15–18).

However, it is optimistic message that many risk factors of metabolic syndrome and course can be modified through lifestyle-based intervention, primarily through changes in diet and physical activity (19). In patients after myocardial infarction, regular 7-days 30-min walking in a park had positive effect on patients’ cardiac function and blood pressure reduction suggesting that CVD prevention through walking in green environments should be encouraged (20). Physical activity, both in the form of aerobic and resistance training, has an important role in the management of hypertension and obesity (21, 22).

In this way physical activity in parks, green space (Nature therapy) produces an opportunity to decrease oxidative stress and inflammation, and risk for obesity, metabolic dysfunctions, and can mitigate the negative effects of hazardous environmental exposures (19, 23, 24). A few recent systematic reviews have reported different effects of aerobic physical training on metabolic syndrome risk factors (25, 26), improved mental health by reducing stress, anxiety, and depression (27). Evidence suggests that physical activity in green spaces can improve health by reducing stress hormones and through the regulation of brain-derived neurotrophic factors (BDNF), enhancing cardiac function, lowering blood pressure, and stress level.

Dietary interventions to prevent or control metabolic syndrome is another possibility. Diets rich in whole grains affect glucose metabolism and improve insulin resistance and insulin secretion (28), consumption of berries (blueberries, cranberries, and chokeberries) may impact biomarkers associated with metabolic syndrome through improving lipid profiles and reducing inflammatory biomarkers (29), while a vegetarian diet combined with aerobic exercise can improve cardiometabolic health biomarkers, which may impact central obesity and metabolic syndrome risk (30, 31). In this way, early identification and management of metabolic syndrome risk factors and biomarkers signaling metabolic disorders, as healthy lifestyle interventions can increase awareness for reducing the risk of CMD.

Although these prior studies highlight the contribution of UFP in metabolic disorders in relation to CVD, no research to date has studied the combined impact of UFP exposure and noise on the development of metabolic syndrome. Within the EU-funded multicenter international MARCOPOLO project, we suggest the METSGREEN cross-sectional study and healthy lifestyle randomized trial. The study aims to investigate the long-term impact of environmental exposures on key indicators of CVD and CMD and estimate whether short-term healthy lifestyle interventions have effect on metabolic disorders biomarkers. The implications of these findings could strengthen the evidence base for pollution-disease associations and provide recommendations for reducing the risk of metabolic disorders, both for public health professionals involved in population health monitoring and for the public.

## 2 Materials and methods

### 2.1 Study design

The METSGREEN study is a part of multicenter international MARCOPOLO study funded by the European Commission through the Horizon Europe program (32). The METSGREEN will be a 48-month longitudinal, single-center study led in Lithuania by Vytautas Magnus University (VMU), with contributions from 15 academic institutions across Europe and North America. The part of the randomized trial will be conducted by researchers at VMU and at the Family Medicine Clinic of Kaunas Clinics at the Lithuanian University of Health Sciences (LSMU). METSGREEN consists of two phases. The first phase Observational Study (cross-sectional) involving 1,000 participants (January 2025–June 2026). The second phase Experimental Clinical Trial (randomized three-arm trial) involving 180 participants. The randomized trial started in October 2025, and the last participant is expected to be enrolled in July 2026. The METSGREEN study will be completed in December 2028. Clinical trial registration NCT ID: NCT07111208. Registered on 08-08-2025.

The study hypothesis is ambient ultrafine particles and noise in the living environment increases the risk of cardiovascular diseases (CVD); however, this risk can be reduced by healthy lifestyle. The METSGREEN seeks to determine the impact of long-term traffic noise and air pollution by UFP on cardiovascular disease and metabolic syndrome risk in humans among 45–64-year-old residents of Kaunas through an observational study. The randomized trial seeks to present evidence-based data for health risk management through short-term lifestyle intervention. Measuring and modeling ground-level ambient monitoring data, we will assess representative sample environmental exposure levels to study effects on prevalence of CVD and the indicators of metabolic disorders: blood pressure, waist circumference, and other health data. During the randomized trial (7-day healthy lifestyle intervention), changes in key clinical indicators of metabolic disorders will be assessed, including waist circumference and blood pressure, high-density lipoprotein (HDL) levels, triglycerides, plasma glucose concentration, as well as novel metabolic and inflammatory indicators that might signal early risk of cardiometabolic disorders. These will be analyzed in relation to environmental exposure, socioeconomic status, and lifestyle factors, assessed by questionnaires. Recommendations will be made to reduce the risk of metabolic disorders.

### 2.2 Objectives and endpoints

Objectives of the Observational Study involving 1,000 participants:

- To measure and model the levels of air pollution and noise exposure in Kaunas, and to determine the relationship between individual exposure and CVD risk, depending on the participants’ social and economic status.
- To assess the long-term impact of road traffic intensity, UFP and noise exposure on key indicators of metabolic disorders.

Objectives of the Interventional Randomized Trial (180 Participants)

- To determine the impact of a healthy lifestyle (physical activity in green spaces and the Mediterranean diet) on the indicators for metabolic disorders.
- To provide recommendations for reducing the risk of metabolic disorders.

Primary Endpoints of Cross-sectional Study: Measured concentrations of UFP and noise levels in 20 locations across the city of Kaunas. Using a land-use regression (LUR) model, exposure levels for 1,000 participants will be modeled and presented linked to health outcomes: prevalence of CVD, blood pressure, waist circumference, and other health data. Traditional clinical CMD risk factors by UFP and noise exposures. Secondary Endpoints of Randomized Trial: Prevalence of key traditional clinical indicators of metabolic disorders, including waist circumference and blood pressure measurements, high-density lipoprotein (HDL) levels, triglycerides, plasma glucose. Short-term intervention effects traditional clinical biomarker changes and metabolic pathway shift in metabolomics, proteomics and DNA methylation biological markers. These will be presented in relation to environmental exposure, socioeconomic status, and healthy lifestyle factors.

Recommendations to reduce the risk of metabolic disorders.

### 2.3 Study population

Participants in the observational component are Kaunas city resident’s male and female, aged 45–64 and live in private apartments. Approximately 1,000 individuals will be randomly selected, excluding individuals in institutional care.

The randomized trial cohort will be drawn from this group and include 180 participants. Inclusion Criteria:

- Age 45–64 years
- Residence in Kaunas City
- Presence of the indicators of metabolic disorder (e.g., increased waist circumference and/or elevated blood pressure)
- Willingness to participate and provide written informed consent
- Agreement to wear a wristband sensor continuously for 7 days

Exclusion Criteria:

- Unstable angina or cardiomyopathy
- Blood pressure >160/110 mmHg
- Neurological diseases or limited mobility
- Presence of a cardiac pacemaker
- Pregnancy
- Alcohol dependence

#### 2.3.1 Justification for sample size

The randomized trial involving 180 participants will assess the impact of a healthy lifestyle on cardiometabolic responses (clinical biomarkers and health risks factors), considering individual UFP exposure levels. To enhance data reliability and reduce the risk of Type I error (false positive) the confidence level is set up at 99.9%. The sample size was calculated using the formula:


$$N=2\times (Z_{1-\alpha /2}+Z_{1-\beta }/\delta 0\;)2\times p\times (1-p),$$

Where:

- N = required sample size
- Z_₁ − ⍺/2_ = 3.29 (for a 0.1% significance level)
- Z_₁ − *β*_ = 0.842 (for 80% statistical power)
- *δ*₀ = effect size = 0.1
- p = expected proportion = 0.3

This gives:

*N* ≈ 2 × (3.29 + 0.842/0.1)^2^ × 0.3 × 0.7 ≈ 58 participants.

To account for potential dropouts (e.g., due to acute infections), a sample size of 60 per group is proposed. Therefore, a total of 180 participants will be enrolled in the randomized trial.

The use of a 99.9% confidence level was intentionally conservative to limit type I error in a multi-arm randomized design with heterogeneous primary outcomes, a strategy recommended when multiple comparisons are inherent to the study structure (33).

The statistical analysis plan will be specified to include linear or generalized linear mixed-effects models for repeated measures between Day 1 and Day 8, as these models appropriately account for within-subject correlation and incomplete longitudinal data (34). Given the large number of secondary and exploratory biomarkers, multiplicity will be addressed using false discovery rate (FDR) control, which is widely recommended for high-dimensional biomedical analyses to balance type I error and statistical power (35, 36). Missing data will be handled using maximum-likelihood–based mixed models or multiple imputation under a missing-at-random assumption, in line with established methodological guidance for clinical and epidemiological studies (37, 38).

### 2.4 Measures to reduce subjective bias

Participants will be randomly assigned to the three clinical trial groups by family physician researchers. Only individuals without listed health exclusions who sign informed consent will be included. Assignment to one of three groups will follow a 1:1:1 ratio. The researcher will handle group allocation and coding. Objective environmental pollution measures and individual residential exposure will be taken. Seven-day monitoring health outcomes include physical activity, sleep disturbances, heart function. Two health checks will include measurements of blood pressure, body composition, waist circumference, body mass index (BMI) and biomarkers. Blood samples (up to 20 mL) will be collected to analyze metabolic biomarkers. All blood samples are anonymized; laboratory personnel and data analysts will be blinded to intervention group assignments. Mathematical modeling will be used during data analysis. It will control for confounding factors to assess the strength of the exposure–health outcome relationship. To reduce potential subjective bias and ensure accuracy and consistency of Mediterranean diet adherence the participants record all food and beverages after each consumption. To validate self-reported physical activity and daily 30-min brisk walks in designated urban green areas, physical activity will be tracked via Fitbit Alta wristband sensors data. During statistical analysis, missing data in Fitbit sensors are handled through clarification of the reason for the data loss, defining “wear time,” and applying appropriate statistical techniques like imputation or deletion.

### 2.5 Biomedical study description

#### 2.5.1 Observational study

A representative sample survey of Kaunas residents is conducted by a certified survey company that complies with strict personal data security, privacy principles, and legal requirements. In accordance with the General Data Protection Regulation (GDPR), data will be securely stored, lawfully processed, and participants will be able to exercise their rights easily.

Approximately 1,000 individuals aged 45–64 will be included based on their responses to an anonymous online survey, which collects self-reported health indicators (physician-diagnosed chronic diseases), socioeconomic status, and basic residential information.

#### 2.5.2 Randomized clinical trial

Participants who completed the online survey and conform to the study inclusion criteria are eligible. Those interested in participating in the healthy lifestyle clinical trial can contact the researchers via the details provided in the internet survey form. Upon contact, participants will be asked to updated information on waist circumference, blood pressure, and address. Eligible participants will be invited to the Family Medicine Clinic of Kaunas Clinics at the Lithuanian University of Health Sciences (LSMU). Only individuals who confirm all inclusion criteria will be included in the randomized clinical trial. Following contact, appointment times will be coordinated. At the initial visit, physician-researchers will review the participant’s health status and check eligibility. Participants will be informed about the experimental clinical trial and an informed consent form.

During the first visit, the participant will be thoroughly informed about:

- The purpose and design of biomedical research
- The methods applied in the study
- The decisions of the ethics committee
- The potential benefits of participation
- The participants right
- Possible risks and inconveniences
- The right to withdraw written consent to participate at any time
- The consequences of withdrawing from the study
- Confidentiality and data protection assurances.

After reviewing this information, the participant will be asked to sign the Informed Consent Form. They will then be randomly assigned to one of three healthy lifestyle groups and instructed on their 7-day health behavior study. Groups: (1) control group (usual routine); (2) physical activity in green space (daily 30-min brisk walk); (3) Mediterranean diet intervention. The health assessment of the participants will be conducted by a family physician on Day 1 and Day 8 using certified medical equipment. Participants will be asked to arrive fasting or having not eaten for at least 2 h before their visits.

Procedures will include:

- Blood sampling (up to 20 mL) for analysis of metabolic biomarkers
- Blood pressure measurement
- Waist circumference measurement
- Body composition analysis

All blood samples are anonymized; laboratory personnel and data analysts will be blinded to intervention group assignments. All participants will wear Fitbit Alta wristband sensors for 7 days, which will collect digital data on physical activity (METs), step count, heart rate, sleep quality. Collected data will be transferred to a secure digital platform and coded to assess the results.

**Figure 1:**
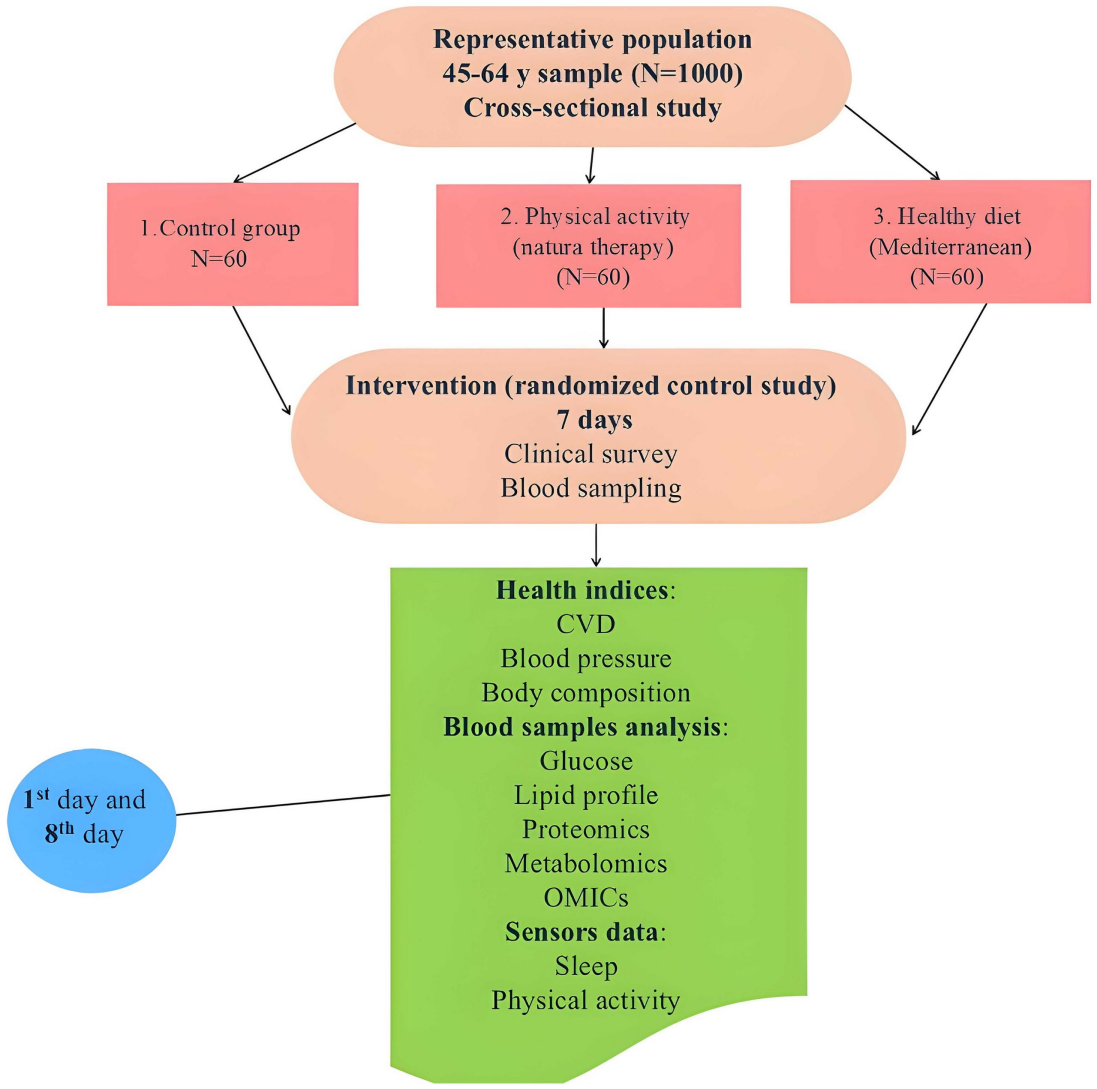
Scheme of the Kaunas Clinical study.

The study plan and phases are shown in Figure 1.

Participants in the intervention arms will be instructed to follow specific Behavioral guidelines over a 7-day period. Green Space Group: Perform daily 30-min brisk walks in designated urban green areas. Compliance will be tracked via Fitbit Alta wristband sensors. Mediterranean Diet Group: Receive dietary counseling and guidance on meal preparation using approved Mediterranean food groups. Nutritional intake will be self-reported and supervised via regular communication with a dietitian. To ensure accuracy and consistency of Mediterranean diet adherence the participants record all food and beverages after each consumption. Control Group: Continue routine daily habits with no Behavioral change. Each group’s clinical health indices and metabolic responses will be assessed by measuring blood pressure, waist circumference, high-density lipoproteins (HDL), triglycerides, plasma glucose levels. To analyze of novel biomarkers for cardiometabolic disorders (proteomic, DNA methylation biomarkers, and inflammatory indicators), anonymized blood samples are sent to the partners laboratories. All data will be analyzed using mathematical modelling to account for confounding variables. Given the short duration of the intervention (7 days), clinical outcomes are limited to traditional clinical biomarkers for cardiometabolic disorders and biochemical markers known to respond acutely. Novel biomarkers for cardiometabolic disorders (omics-based outcomes – metabolomics, proteomics, epigenetic markers) are treated as exploration and hypothesis-generating, aimed at identifying early molecular signatures rather than short-term interventional study clinical effects. Results will substantiate the impact of UFP and noise exposure on the biomarkers of metabolic disorders and present recommendations to healthcare professionals and the public targeted for reducing metabolic disorder risks.

#### 2.5.3 Biomarker analysis and evaluation

Collected blood samples will be first analyzed for Clinical Metabolic Indicators: fasting plasma glucose, triglycerides, HDL cholesterol. Then, biological samples for metabolic response will be processed and analyzed at partner institutions. Inflammatory Markers: selected cytokines and proteins via immunoblotting; Advanced Omics Profiling: targeted metabolomic, proteomic, and epigenetic analysis. DNA methylation at the National Center of Pathology, Laboratoire National de Santé (Luxembourg); Proteomics at the University of Southern Denmark; Metabolomics at the University of Eastern Finland. The analysis of novel biomarkers for cardiometabolic disorders primarily uses advanced analytical chemistry techniques, particularly mass spectrometry (MS) and Nuclear Magnetic Resonance (NMR) spectroscopy, coupled with chromatography for sample separation. Primary proteomics screening comprises of 4–6 oxidative stress and inflammation markers via immunoblotting. In the second phase, based on the initial results, advanced OMICs analysis will be conducted using high-resolution techniques, such as redox/phospho-proteomics, metabolomics, DNA methylation via EPIC chip (for genome-wide methylation analysis). Missing data will be handled using maximum-likelihood–based mixed models or multiple imputation under a missing-at-random assumption, in line with established methodological guidance for clinical and epidemiological studies (37, 38). All data will be analyzed using mathematical modelling to account for confounding variables. During the statistical analysis, we will use a standardized exposure-health response analysis approach to analyze the data from the observational cross-sectional study ant the interventional randomized study. Key methods: multivariate logistic regression analysis, adjustment for confounding factors using mathematical modelling to estimate the strengths of the relationship and 95% confidence intervals between the variables.

#### 2.5.4 Ethical considerations

The Kaunas Regional Biomedical Research Ethics Committee has granted approval for this study (03-07-2025, approval number: BE-2-43). The project follows international ethical principles for research with humans Declaration of Helsinki, revised in 2008. Participation is voluntary, with informed consent required for the randomized trial. Participants may withdraw at any time and have the right to access, correct, or withdraw their data. Minimal risks are anticipated, limited to standard blood sampling. All personal data are handled according to the General Data Protection Regulation (GDPR). Participants are identified in study documents (excluding the consent form) by assigned code, linkable to identity. Personally identifiable data are stored separately and securely, accessible only to the principal investigator and designated team members.

## 3 Discussion

Nowadays research continues to show particulate matter exposures health impacts, and the precautionary principle asks politicians to take preventative action to protect the environment and public health even if scientific evidence is not fully conclusive. Prior studies also highlight the contribution of UFP in metabolic disorders in relation to CVD, however, no research to date has studied the combined impact of UFP exposure and noise on the development of the metabolic syndrome. This research, using cross-sectional and interventional studies will fulfill this gap by investigating environmental exposures associations with traditional clinical biomarkers and novel biomarkers for cardiometabolic disorders, and strengthen the evidence base for pollution-disease associations. In this study, use of healthy lifestyle and Nature-based interventions such as physical activity in green spaces are promising strategies to counteract the health effects of ambient pollution, and the clinical metabolic biomarker results presents data for increasing population knowledge about healthy lifestyle impact on CMD health.

Prior studies showed that higher physical activity in green environment, improved diets and psychosocial stress management may have positive impacts on CVD risk and metabolic syndrome course (19). A randomized trial showed that in patients after myocardial infarction, regular 7-days 30-min walking in a park had a greater positive effect on patients’ cardiac function and blood pressure reduction than walking in an urban environment, suggesting that CVD prevention through walking in green environments should be encouraged (20). A systematic review and meta-analysis indicate that physical activity at moderate intensity during leisure time has a significant effect on lowering blood pressure compared to a non-intervention control group (39).

According to the joint recommendation of international scientific societies on the clinical diagnosis of metabolic syndrome, the key diagnostic criteria include central obesity, elevated blood pressure, increased plasma glucose levels, elevated triglyceride levels, and low high-density lipoprotein (HDL) cholesterol levels (40). Because early identification and management of metabolic syndrome risk factors can significantly reduce the risk of CVD and fatal outcomes (41), we will use these international criteria for hypothesis substantiation. Some studies presented blood biological markers associations with cardiometabolic disorders, as well as blood pressure and the prevalence of hypertension (42). So, managing metabolic syndrome with a healthy lifestyle involves physical activity in green environment, healthy diet, and psychosocial stress management (43, 44), which may have effect on biomarkers for metabolic disorders.

Novel biomarkers for cardiometabolic disorders, epigenetic signatures, including DNA methylation, are increasingly recognized as responsive to lifestyle factors such as diet and physical activity, with locus-specific methylation changes linked to metabolic regulation (45). Metabolomics approaches are progressively used to capture molecular signatures reflective of diet and metabolic health even over short exposures (46). Proteomic studies demonstrate rapid proteomic responses to physiological stimuli; exercise alters circulating proteins tied to immune and metabolic pathways acutely (47). Recent human studies also show that short-term dietary interventions can induce measurable metabolomic changes within days, such as plasma metabolite profile shifts after a 6-day Mediterranean diet intervention (48). In this study, analyzing short-term intervention we differentiate clinical endpoints from exploratory omics endpoints, which are used for hypothesis generation aimed at identifying early molecular signatures rather than short-term interventional study clinical effects.

Nature-based interventions such as physical activity in green spaces are a promising strategy to counteract the health effects of ambient pollution. Regular exposure to nature has been shown to improve autonomic nervous system balance, reduce cortisol levels, and promote cardiovascular resilience. Dietary interventions, particularly the Mediterranean diet, rich in unsaturated fats, antioxidants, and anti-inflammatory nutrients, are also recognized for their cardioprotective effects. Despite this, very few studies integrate objective environmental exposure assessments with clinical lifestyle interventions and biomarker-based outcomes of metabolic disorders. The results of this study will support the effects of UFP and noise exposure on the clinical biomarkers of metabolic disorders and present recommendations to politicians and healthcare professionals for reducing metabolic disorder risks.
